# Construction and Compensation of a Dimensional Accuracy Model of a Powder Bed via Laser Sintering

**DOI:** 10.3390/polym15163417

**Published:** 2023-08-16

**Authors:** Jiaming Dai, Yanling Guo, Jian Li, Shuai Guo, Deyu Meng, Haoyu Zhang, Yifan Guo

**Affiliations:** College of Mechanical and Electrical Engineering, Department of Mechanical Engineering, Northeast Forestry University, Harbin 150040, China

**Keywords:** laser sintering, powder bed dimensional accuracy, polymer, compensation model

## Abstract

In the laser sintering (LS) printing process, a printed part is formed by sintering layer-by-layer on the powder bed. Thus, it is necessary to consider the dimensional accuracy of the laser-sintered powder bed as an important evaluation index. In this paper, a generalized powder bed–size accuracy compensation model is proposed for non-crystalline thermoplastic polymer materials. Taking polyethersulfone (PES) material as an example, the main factors influencing powder bed dimensional accuracy during LS printing are modeled and analyzed experimentally in this study, including four important factors: laser reference deviation, temperature deviation, density deviation, and secondary sintering deviation. In this study, CX_A200 LS equipment is used for prototyping and verification, a 3D scanning method is used to measure the printed parts, and the measurement results are digitally compared and analyzed. On this basis, the relationship of each influencing factor in the proposed compensation model is determined experimentally, and the experimental results demonstrate that the proposed compensation model is approximately 95% effective in terms of correcting the deviation of powder bed dimensional accuracy.

## 1. Introduction

Additive manufacturing (AM) is an advanced manufacturing technology that has irreplaceable and unique advantages in contemporary medical, aerospace [1], and high-end manufacturing industries [2,3,4,5]. Laser sintering technology (LS), as an additive manufacturing technology [6,7,8], has a unique advantage in the forming of polymer materials [9]. In contrast to the traditional forming process of equal or reduced materials, laser sintering technology is formed by the continuous accumulation of layer-by-layer sintering on a powder bed [10]. In the process of layer-by-layer sintering, the dimensional accuracy of the parts is affected by many factors, and it is not ideal. In recent years, with the development of laser sintering technology, and although the research heat is higher in the aspects of materials [11,12] and numerical simulation [13], the importance of the dimensional accuracy of parts has gradually increased [14].

In related fields, Pil et al. [14] reviewed research on process dimensional accuracy in AM, and they argued that high dimensional accuracy is one of the main scientific challenges for additive manufacturing in order to improve product quality. Huang et al. [15] have proposed to introduce CAE simulation into the rapid prototyping process and pre-process CAD models with dynamic reverse compensation in order to improve machining accuracy. Therefore, this paper focuses on the construction and compensation of the precision model.

Specifically, in the field of laser sintering, Jian et al. [16] studied dimensional shrinkage deformation in the direction of forming materials. Li [17] et al. studied the shrinkage prediction of the model regarding numerical simulation, and they proposed a compensation method. Dotchev et al. [18], meanwhile, discussed a method to improve dimensional accuracy, and argued that the shrinkage in the X and Y directions was linear and that the method of constant proportional factor was used to compensate, whilst the workpiece with guaranteed accuracy within IT13 was obtained. However, compared with the corresponding machining field, IT13 is not competitive, which means that there is still room for improvement in the size accuracy of the powder bed. 

Stopp et al. [19] analyzed the correlation between printing direction, position, and accuracy in different regions, and they believed that the position had a great impact on accuracy. Brajlih et al. [20] mentioned the effect of inertia of the scanning mirror and positioning error when discussing shrinkage problems. Yan et al. [21], meanwhile, discussed the influence of the secondary sintering phenomenon on laser sintering accuracy. Therefore, this study chooses to discuss the comprehensive dimensional accuracy of the interaction between the size accuracy of the powder bed, the shrinkage problem, the secondary sintering problem, and the spot positioning accuracy.

Gaurav et al. [22] argued that the geometric freedoms associated with AM create new challenges in maintaining and communicating the dimensional and geometric accuracy of the parts that are produced. The influence of the AM process on current geometric dimensions and tolerances (GD&T) practice is reviewed. Therefore, in the process of measuring the size accuracy of the powder bed, the three-dimensional measurement method is used to check the size result. 

In summary, the dimensional accuracy problems generated in laser sintering technology are accumulated layer-by-layer, and so this paper focuses on the scale of the powder bed. We analyze and model the dimensional accuracy of powder bed printing results, and we propose a powder bed dimensional accuracy compensation model. Finally, we carry out an experimental verification of the accuracy compensation model, and we analyze and discuss the influence of the related results.

## 2. Compensation Model Creation

In this section, the sources of influence on the creation of the powder bed dimensional accuracy model are analyzed in order to determine the limited influence range of relevant factors on the dimensional accuracy of a powder bed. In addition, the principle of the proposed accuracy compensation model is introduced, and the method employed to obtain the parameter terms involved in the accuracy compensation model is also expanded on.

### 2.1. Analysis of Sources of Influence on Powder Bed Dimensional Accuracy

The analysis of powder bed dimensional accuracy has special significance for LS technology because the LS printing process constitutes continuous accumulation of layer-by-layer sintering in the powder bed, and, therefore, the fundamental accuracy of the powder bed has a considerable impact on the overall accuracy analysis of LS technology [23].

**Figure 1:**
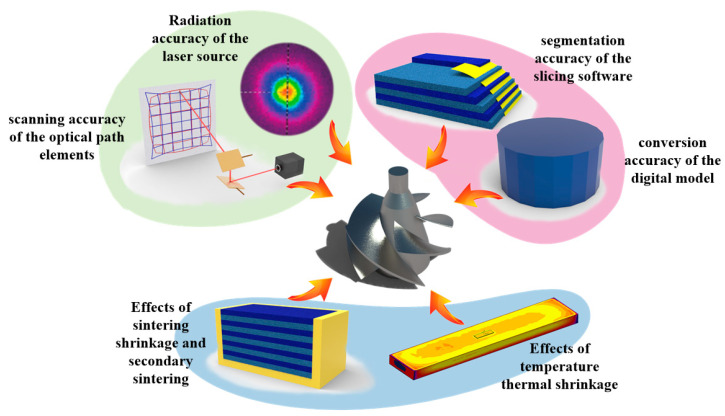
Sources of influence on the dimensional accuracy of printing surface.

The sources of influence on powder bed dimensional accuracy include the accuracy of the reference laser path, the accuracy of the digital model, and, lastly, the physical environment of the process. Among these sources, the effect of the reference laser path includes the effect of the laser scanning accuracy and the quality of the radiation accuracy of the laser source [24]. The effect of the digital model accuracy includes the conversion accuracy of the digital model as well as the segmentation accuracy of the slicing software [25]. In addition, the physical environment can affect temperature shrinkage [26], sintering shrinkage, and secondary sintering [27,28] (see Figure 1).

The effects of these influencing factors are summarized as follows. In terms of the effect on the reference laser path, the minimum positioning accuracy of the scanning vibrating mirror control unit can generally reach 5 mrad and the height of the scanning vibrating mirror and the target surface is generally between 300–500 mm; thus, the laser sweep equivalent positioning accuracy error can be estimated as approximately 0.137–0.08 mm, which is within the range of the ideal precision powder bed thickness. However, without effective correction, the integrated accuracy error caused by the assembly of the scanning vibrating mirror control unit and other static optical elements makes the spot positioning accuracy of the powder bed surface decrease greatly. The local error of laser sintering equipment used in this paper can reach 4 mm, and this is beyond the ideal accuracy range. Therefore, this paper ignores the positioning accuracy of the scanning vibrating mirror control unit itself, but it does not ignore the positioning accuracy of the laser spot.

The quality of the radiation accuracy of the laser source is generally more stable. Typically, the laser is focused after radiation on the target work surface, and the spot size is generally between 0.2–0.8 mm. In addition, the laser illuminates the powder bed positively and obliquely in order to produce aberrations in the shape of the spot, and this spot error is influenced by the deflection angle θ of the optical path. The scanning limit angle of the scanning vibrating mirror is generally ±15°, as inferred from the laser light source radiation accuracy deviation in θ = ±15°, in order to reach the limit deviation, i.e., approximately 0.0071–0.028 mm. This parameter can be predicted by the 3D geometric relationship, so relatively random influence is small and easy to handle in the control program. Therefore, the proposed compensation model ignores the influence of the quality of the radiation accuracy of the laser source.

The conversion accuracy of the digital model is set according to digital model protocol and the selected mode, and it can usually reach about 0.01~0.0001 mm. Generally, the conversion accuracy of the layer is taken to be 0.001 mm, which is a level of accuracy that far exceeds the deviation in other areas. The conversion accuracy of the digital model can thus be negligible.

Generally, the segmentation accuracy of slicing software is affected by the slice thickness and the spot size. The general molding thickness of the polymer material targeted in this study is approximately 0.08–0.2 mm, and the corresponding resulting contour deviation is within 0.057–0.14 mm. In addition, the interlayer segmentation accuracy can be estimated accurately using geometric methods based on parameters; therefore, the accuracy impact exhibits enhanced stability, and it is typically improved via post-processing techniques. As a result, the proposed compensation model ignores the segmentation accuracy of slicing software.

The effect of temperature shrinkage is reflected by the process temperature change, and the general range is within 60–300 °C. Generally, the size range of the printing parts is within 500 mm. According to the estimation of the typical linear expansion coefficient in the order of $a\times 10^{-5},(a\in \left[1\sim 10\right])$, the impact accuracy of temperature thermal shrinkage is affected by the size of the fabricated parts, and its impact range is generally 1 × 10^–5^ mm–15 mm. Hence, the proposed compensation model considers the effect of temperature shrinkage.

The main source of effect on sintering shrinkage is the variation in the density of the target material, and it is difficult to determine the specifics of sintering shrinkage accurately because material properties vary and the effect of temperature varies for different materials. In addition, there is an increasing demand for high-temperature material applications, e.g., PEEK materials require preheating of up to 300 °C [29]. Hence, the proposed compensation model considers the effect of sintering shrinkage.

The primary cause of secondary sintering is the absorption of energy from the material in the non-sintered region for solidification, which leads to the influence of the accuracy of secondary sintering by the temperature field, typically up to 0.0001–0.2 mm. Eradicating the secondary sintering problem for non-crystalline thermoplastic materials is a challenging task, and the proposed compensation model thus considers the influence of secondary sintering.

**Figure 2:**
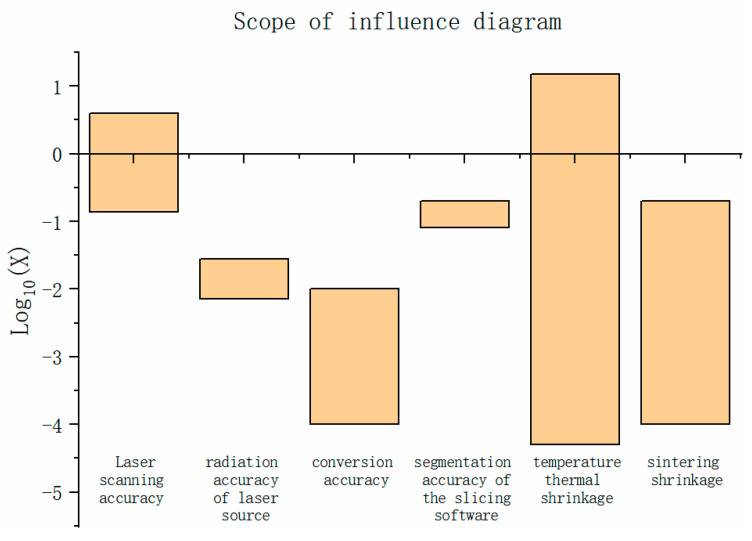
Schematic diagram of the order of magnitude of each factor.

The effect of these factors on the dimensional accuracy of the powder bed is shown in Figure 2, where the results take 10 as the bottom log plot. As is evident in Figure 2, the laser light source radiation and the conversion accuracies of the digital model are much higher than the other factors, and the order of magnitude of the influence on the accuracy is much higher than the requirement of this paper on the size accuracy compensation model of the powder bed. Thus, it is reasonable to ignore the problems related to these two factors. Thus, the laser scanning deviation, the temperature deviation, the density deviation, and the secondary sintering deviation are the primary foci in the proposed powder bed dimensional accuracy compensation model in this paper.

### 2.2. Accuracy Compensation Model

(1) Accuracy model

The accuracy model developed based on the above influencing factors is expressed in Equation (1):

(1) $$L_{∆}=L-L_{meas}=\omega \cdot {∆}_{Las}+{\alpha \cdot ∆}_{T}+{\beta \cdot ∆}_{\rho }-\gamma \cdot {∆}_{SS}$$

The variables related to the mathematical model are described in (Table 1).

In this paper, ${∆}_{Las}$ (laser reference deviation term) will be determined by grid sintering calibration, and it is given in Section 2.3.

For ${∆}_{T}$ (temperature deviation term), this paper is based on the joint estimation of the linear expansion coefficient of the PES solid material combined with the temperature deviation and the dimensions of the molded part, as modified by the coefficient α · ${∆}_{T}$, is described in detail in Section 2.4.

For ${∆}_{\rho }$ (density deviation term), this paper will be measured by density experiments and revised with a coefficient β · ${∆}_{\rho }$, and is described in detail in Section 2.5.

For ${∆}_{SS}$ (secondary sintering deviation term), this paper will be determined by thickness increment experiments, and the factor γ is revised. ${∆}_{SS}$ is described in detail in Section 2.6.

Equation (1) is used to relate the four core deviation terms to the ideal size L and the actual size $L_{meas}$ by means of the revision factor in order to obtain the accuracy compensation model. Subsequently, scan path planning is performed in order to generate process files that can be read by the LS device, as described in Section 3.1, and finally printed by the LS device.

(2) Compensation method

Currently, the LS molding process generally begins by exporting a generic mesh surface file, such as a standard template library (STL) file, from a Computer Aided Design (CAD) model file of the finished product. Further slicing is then performed using slicing software, such as Magics and Cura. Then, scan path planning is performed in order to generate process files that can be read by the computer device and printed by the LS device. These workflows are generally undertaken on computers. The accuracy control of this process is performed on $L_{∆}$, but it is based on the objective existence of deviation terms and various parameters, e.g., forming size, forming temperature, and change material. At present, there is no recognized effective precision control means to comprehensively deal with the problem of plane dimension accuracy. 

Equations (2) and (3) are expressed as follows:

(2) $$L_{meas}=L-{\omega \cdot ∆}_{Las}-{\alpha \cdot ∆}_{T}-{\beta \cdot ∆}_{\rho }+\gamma \cdot {∆}_{SS}$$

(3) $$L={L_{meas}+\omega \cdot ∆}_{Las}+{\alpha \cdot ∆}_{T}+{\beta \cdot ∆}_{\rho }-\gamma \cdot {∆}_{SS}$$

The ideal value L in Equation (3) is replaced by the demand dimension L_C of the CAD model, and L_meas is taken as the input quantity L to obtain Equation (4).

(4) $$L_{C}={L+\omega \cdot ∆}_{Las}+{\alpha \cdot ∆}_{T}+{\beta \cdot ∆}_{\rho }-\gamma \cdot {∆}_{SS}$$

In Equation (4), L is the input parameter and $L_{meas}$ is the output result. The compensation model can be obtained by adjusting the input-output relationship of $L_{meas}$ and L. It can be understood that in order to obtain an ideal result, such as $L_{meas}$, it is necessary to input parameter $L_{C}$ after compensation model correction. In the final printing process, $L_{C}$ is taken as the input parameter to carry out relevant processing on the computer and finally obtain the ideal result size L. 

### 2.3. Purpose and Significance of Laser Scanning Deviation Experiment

For LS devices, the distortion of the optical scan path is caused by assembly problems, optical structure problems, and optical lens problems. Although common devices possess optical control system deviation correction in order to improve the optical aberration problem [30], in practice, optical scan path distortion is also influenced by the specific working environment, temperature changes, and other printing device characteristics. 

**Figure 3:**
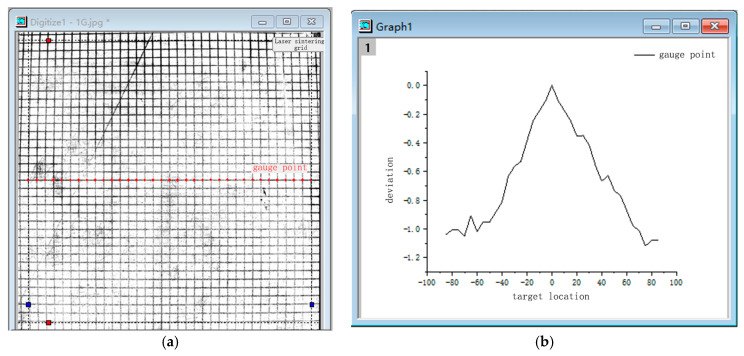
Planar grid sintering and digital information extraction: (**a**) laser sintering grid; (**b**) extracting digital information extracted from the gauge point.

Some deviation between the ideal target point and the actual target point remains, and this directly affects the dimensional accuracy of the printed part in single-layer molding. The ideal target point is the identification node in the ideal laser path, and the actual target point is the identification node in the actual laser path. This deviation can further affect the overall dimensional accuracy of the printed part via layer-by-layer accumulation. Here, laser-tuned photo paper was utilized to print a single plane grid and perform a plane scan in order to obtain digital information about the optical basis deviation of the powder bed (Figure 3). After scanning, a calibration analysis was performed using Origin2018 software in order to determine the relationship between the input parameter L and the laser scan deviation ${∆}_{Las}$.

### 2.4. Determining Temperature Deviation

Using the basic definition of the linear expansion coefficient to obtain ∆_T, the linear expansion coefficient of PES material parameters is taken as $2.3\times 10^{-5}/℃$ in this paper [31], and the temperature variation can be obtained based on the actual printing parameters with a preheating temperature of 89 °C, from which Equation (5) is obtained. Finally, the relationship between the temperature variation parameter ${∆}_{T}$ and input parameter L can be determined as follows:

(5) $${∆}_{T}=2.3\times 10^{-5}\times (89-22)\times L$$

### 2.5. Purpose and Significance of Density Measurement Experiment

In the printing process, the material should ideally fill the entire molding cavity, and, therefore, the overall volume and the mass of the material inside the cavity are constant. However, the density of the material in the powder state is significantly different from the density of the ideal dense part; therefore, a volume change inside the molding cavity will occur due to the conservation of mass, which is the root cause of the impact of density change on accuracy. This involves three main factors, which are described below.

The first factor is that due to the difference in surface temperature between the sintered and non-sintered areas of the fabricated part during the powder spreading process, the relative high-temperature area above the powder mobility decreases, the porosity decreases, and the stacking density increases, which results in an increased relative material mass. 

The second factor is that the sintered area is subject to downward shrinkage collapse. In other words, a certain amount of shrinkage occurs in each layer during the process. Due to the friction between the adjacent parts and the powder, the previous forming layer will suppress the planar shrinkage of the current forming layer while there is nearly no suppression of the longitudinal direction, and, thus, the longitudinal shrinkage of each layer is dominant. 

The third factor is that the parts end up with more pores and residual internal stresses, which inhibits the density shrinkage of those parts.

**Figure 4:**
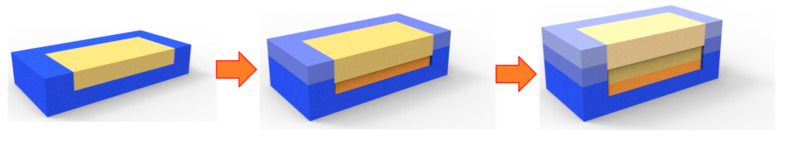
Layer shrinkage.

Due to these three factors, the actual amount of powder spread in the sintering area will be somewhat greater than the theoretical value, thereby reducing the gap between the material quality in the forming area before and after sintering in the actual forming process, and thus suppressing the density shrinkage of the material to a certain extent. As a result, the material shrinkage due to density change is not obvious (Figure 4).

The change in density affects the shrinkage of the material and, therefore, the dimensional accuracy. In this study, a density measurement experiment was designed in order to find the relationship between the effect of density change and the print size. Throughout the LS process, if the liquefaction process of the material transient is ignored, the state of the material at the macroscopic level can be simply divided into powder and monolithic block. For this purpose, the material must undergo the following three processes, which correspond to three density states:

(1) The initial addition process from the outside into the powder supply box corresponds to the powder supply stacking density $\rho_{0}$.
(2) The powder spreading process transferred from the powder supply box to the forming box corresponds to the powder spreading density $\rho_{1}$.
(3) The sintering process in the forming box to form an entire block corresponds to the density of the part $\rho_{2}$.

**Figure 5:**
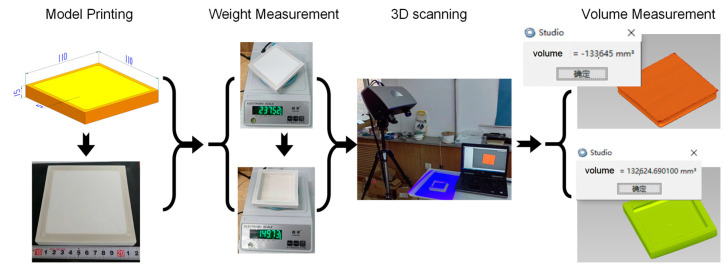
Determination of bulk mass and volume.

The empty box with determined volume $V_{0}$ is manually filled with powder and compacted to measure the mass $m_{0}$, and the stacked density of the powder supply is calculated as $\rho_{0}=m_{0}/V_{0}$ as $\rho_{0}$. As shown in Figure 5, the stacked density $\rho_{1}$ and the part density $\rho_{2}$ should be measured using the open-ended empty box printed parts, which requires the number of layers of the part without posterior powder laying. First of all, the excess powder at the bottom of the four walls of the printed parts is removed, and the overall weight $m_{t}$ is measured on the printed parts. Further 3D measurement of the cleaned parts was then carried out in order to obtain the volume $V_{1}$ of the powder inside the empty box and the volume $V_{2}$ of the parts. Finally, the corresponding density was calculated as $\rho_{1}=(m_{t}-m_{2})/V_{1}$ and $\rho_{2}=m_{2}/V_{2}$. From an experimental perspective, the material volume and the mass are functions of input parameter L; hence, the relationship between ${∆}_{\rho }$ and the input parameter L can be obtained.

Because the 1.5 powder supply multiplier in the experimental process of this paper is problematic, it is based on the conservation of mass, as shown in Equations (6)–(9).

(6) $$V_{bef}\cdot \rho_{1}\leq V_{aft}\cdot \rho_{2}\leq V_{bef}\cdot {1.5\rho }_{0}$$

(7) $$\frac{V_{aft}}{V_{bef}}=\frac{X_{aft}}{X_{bef}}\cdot \frac{Y_{aft}}{Y_{bef}}\cdot \frac{Z_{aft}}{Z_{bef}}$$

(8) $$\frac{X_{aft}}{X_{bef}}\approx \frac{Y_{aft}}{Y_{bef}}\approx 1-\frac{{∆}_{\rho }}{L}$$

(9) $$C_{1}\cdot \frac{\rho_{1}(L)}{\rho_{2}}={\left(1-\frac{{∆}_{\rho }}{L}\right)}^{3}\cdot C_{0}$$

Here, $V_{bef}$ is the powder provided before powder laying, and $V_{aft}$ is the actual powder filled into the powder bed after powder laying. The value of ${∆}_{\rho }$ is taken as a function of the length dimension of the part in Equation (9), where a Z-directional revision factor $C_{0}$ is introduced, because the effect of the plane scale X; where the *Y*-axis direction differs from that of the *Z*-axis direction; and where a density correction factor, $C_{1}$, is introduced. The relationship between the ∆_ρ term and the input parameter L can be determined as follows:

(10) $${∆}_{\rho }=-1\times L\cdot \left(1-\sqrt[{3}]{\frac{\rho_{1}\left(L\right)}{\rho_{2}}}\right)$$

### 2.6. Secondary Sintering Deviation Printing Experiment

Secondary sintering is primarily caused by the temperature gradient of the material during the printing process, which causes some of the material in the non-sintered area to absorb energy and solidify on the surface of the printed part, and it has certain mechanical properties that are difficult to remove. This reduces the dimensional accuracy of the printed part.

**Figure 6:**
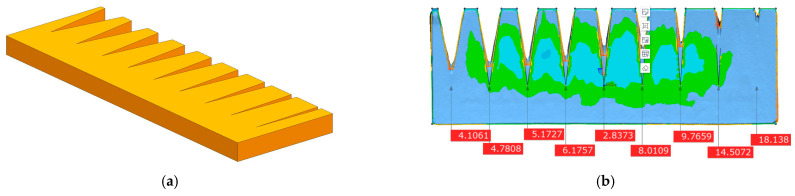
Inner corner sample model and sintered part inspection: (**a**) ideal print, (**b**) detection and calibration of printed parts.

**Figure 7:**
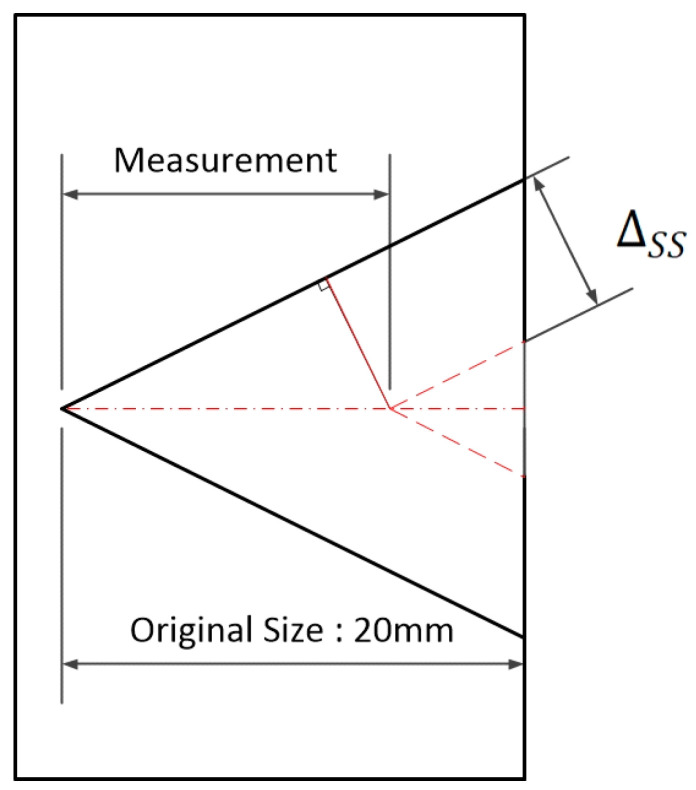
Depth deviation diagram.

The thickness of this hard-to-remove layer is difficult to measure by directly stripping peeling, and an inner corner forming deviation experiment was conducted to obtain the equivalent thickness of the solidified layer and, thus, the secondary sintering deviation. As shown in Figure 6, the inner angle sample is printed and inspected. The exact depth dimension of the angular sample is obtained by 3D scanning to convert the equivalent secondary sintering thickness ${∆}_{SS}$ (Figure 7). 

## 3. Accuracy Compensation Model Variable Acquisition Experiments

Experiments conducted to acquire the variables of the proposed accuracy compensation model are described, and the corresponding results and the functional relationships are derived, following which the complete compensation model for the dimensional accuracy of the powder bed is obtained.

### 3.1. Material Parameters and Equipment

**Figure 8:**
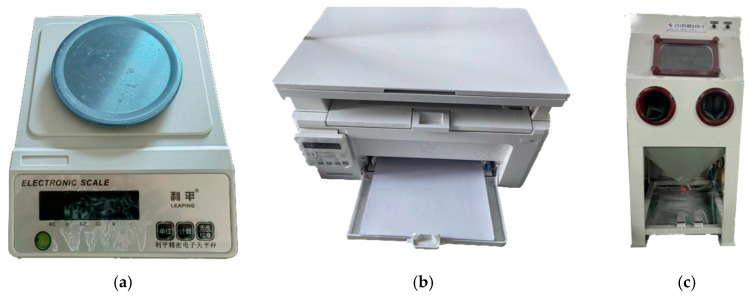
Auxiliary equipment: (**a**) electronic scale; (**b**) flatbed scanner; (**c**) sandblaster.

**Figure 9:**
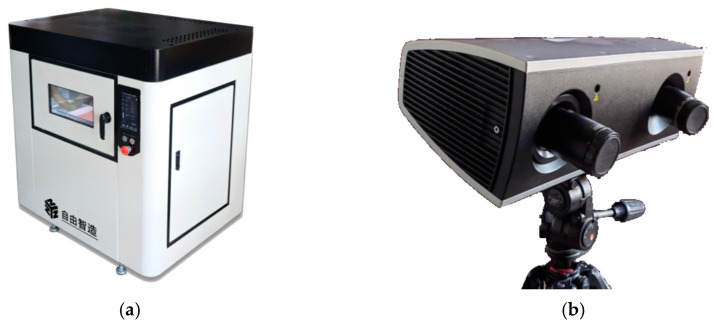
Laser sintering equipment and 3D measurement equipment: (**a**) CX_A200 laser sintering equipment; (**b**) Colin3D scanner.

Figure 8 shows the experimental materials and equipment, including the PES molding material, the laser thermal photo paper, the LS molding equipment, the 3D scanning and inspection equipment, the flat scanner, the precision electronic scale, and the manual sandblasting machine. The experimental material used is PES powder, which was provided by Anhui Tian Nian Materials Technology, Ltd., with a particle size of 100–120 mesh. The LS equipment utilizes CX_A200 nonmetal LS equipment from Harbin Free Wisdom Technology Development Co., Ltd. (as shown in Figure 9a), and the detailed parameters are provided in Table 2. The 3D scanning device used in this study is a Colin3D scanner (Figure 9b), and the detailed parameters are given in Table 3. The abrasiveness of the sandblasting equipment is 100 mesh white corundum.

### 3.2. Acquisition of ${∆}_{Las}$ Terms

**Figure 10:**
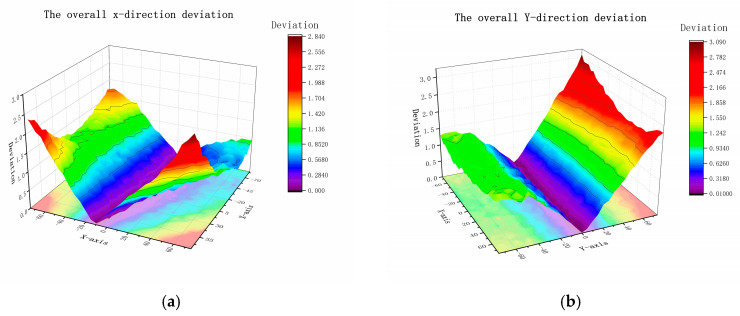
Forming surface scanning grid deviation: (**a**) X-direction deviation; (**b**) Y-direction deviation.

The digitized results of the deviation of the grid results scanned by the laser through the vibrating mirror system are shown in Figure 10:

As is evident in Figure 10, a default dimensional deviation exists in the LS equipment that affects the base dimension of the part and causes the deviation. From Figure 10, the deviation function ${∆}_{Las}\left(x,y\right)$ based on the forming center can be obtained, and it can be simplified to obtain ${∆}_{Las}\left(L\right)$ based on the axial dimensional deviation of the part relative to the center according to the overall X- and *Y*-axis averaging trend.

**Figure 11:**
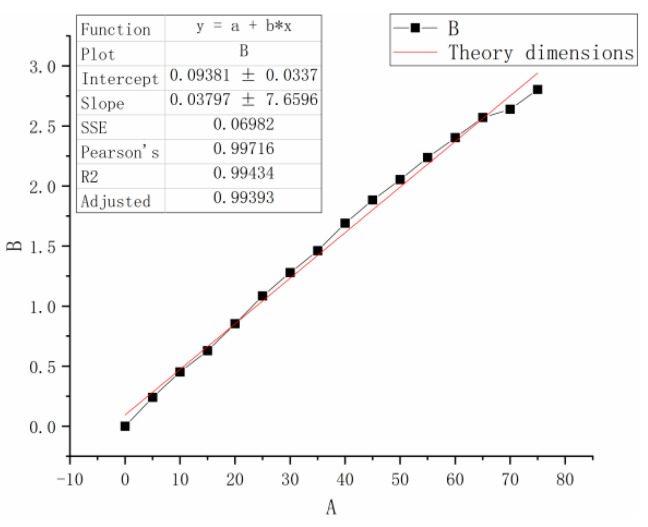
X-direction deviation fitting curve.

To simplify the calculation, the X-direction is considered as the length direction of the actual part based on the characteristics of the part, and the width direction is taken as 20 mm with relatively small influence. Here, ${∆}_{Las}\left(L\right)$ is used as the deviation function (Figure 11). The relationship used to describe the influence of the scanning vibrating mirror system on the accuracy is obtained using the fitting method expressed in Equation (11):

(11) $${∆}_{Las}=0.09381+0.03797\times L$$

### 3.3. Acquisition of ${∆}_{\rho }$ Term

**Figure 12:**
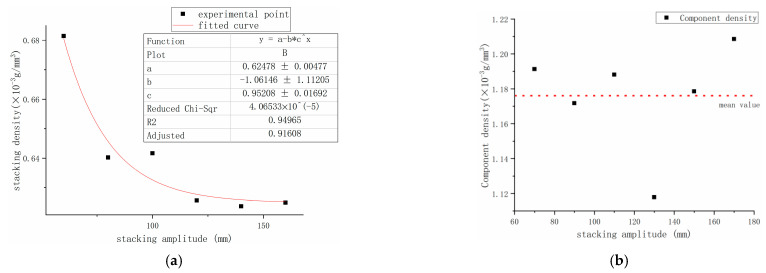
Experimental results of powder laydown density and part density: (**a**) pavement powder accumulation density $\rho_{1}$; (**b**) fabricated parts’ density $\rho_{2}$.

The supply powder stacking density $\rho_{0}$ is shown in Table 4, and the corresponding experimental results for the powder bed stacking density $\rho_{1}$ and the part density $\rho_{2}$ are shown in Figure 12:

The variance of the mean value in Table 4 is 0.000273, and the density of the donor powder can be considered $\rho_{0}=0.735\times 10^{-3}(g/{mm}^{3})$.

From Figure 12, the powder bed stacking density $\rho_{1}$ decreases with the increase in the bulk area, and it tends to a fixed value. This can be attributed to the fact that the effect of shrinkage is more pronounced for small parts and that the shrinkage of the small parts after molding may be more substantial for the increment of the test density, thereby closing the gap between powder density and powder supply stacking density following the spreading of the powder. At larger sizes, the powder rolls are not densely pressed, and some of the powder at the molding boundary will be absorbed by the molding area; thus, generally, as the stacking width increases, the stacking density $\rho_{1}$ tends to a stable limit. Here, the density $\rho_{2}$ of the fabricated parts was more uniformly dispersed around $1.176\times 10^{-3}(g/{mm}^{3})$ with a variance of 0.004839 and a 95% confidence interval of [1.12034, 1.23166] $\times 10^{-3}(g/{mm}^{3})$.

In summary, the constants $\rho_{0}=0.735\times 10^{-3}(g/{mm}^{3})$ and $\rho_{2}=1.176\times 10^{-3}(g/{mm}^{3})$ were taken in order to obtain the relationship expressed in Equation (12):

(12) $${∆}_{\rho }=-L\cdot \left(1-\sqrt[{3}]{\frac{C_{1}\cdot \left(0.62478+0.1614\times 0.95208^{L}\right)}{C_{0}\cdot 1.176}}\right)$$

### 3.4. Acquisition of ${∆}_{SS}$ Terms

**Figure 13:**
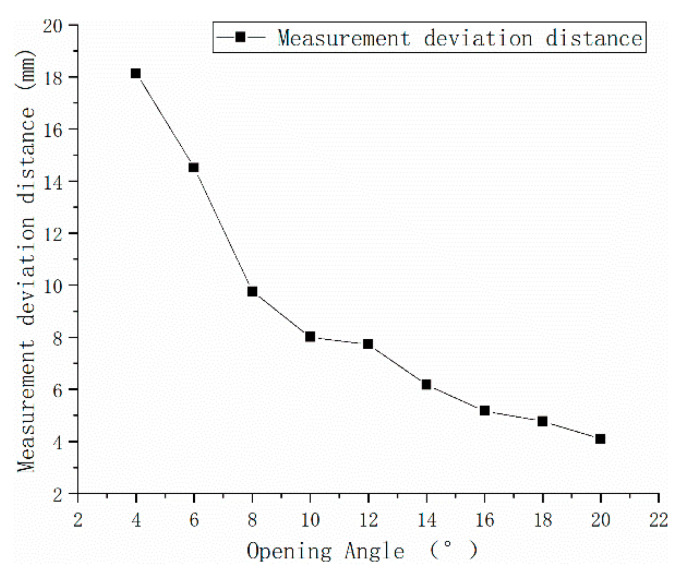
3D analysis value with an angle change line graph.

**Figure 14:**
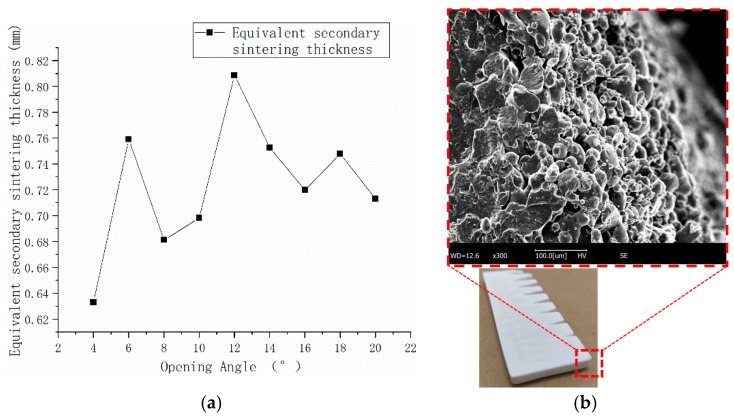
Results of secondary sintering experiment: (**a**) secondary sintering thickness for equivalent results; (**b**) secondary sintering electron microscope.

The experimental results of the inner corner forming deviation are shown in Figure 13, and the equivalent thickness of the secondary sintering is converted according to what is shown in Figure 14:

The average thickness of the secondary sintering obtained by conversion is 0.723753 mm, the variance is 0.020712, and the 95% confidence interval is [0.7102~0.7373], so ${∆}_{SS}$ can be taken as a constant. Because it is difficult to clean powder at sharp corners under experimental conditions, the measurement of the secondary sintering thickness may not be accurate enough, so the revision coefficient γ is used to make a certain revision. Through electron microscopy, it can be clearly identified that the left side is a good sintering zone, and that the material is well sintered and melted. The material in the right boundary region can be in an obvious state, which is the secondary sintering zone, and the secondary sintering thickness of the corner is relatively thin. The final value of ∆_SS_ is taken as a constant 0.723753 mm.

### 3.5. Powder Bed-Size Accuracy Compensation Model

**Figure 15:**
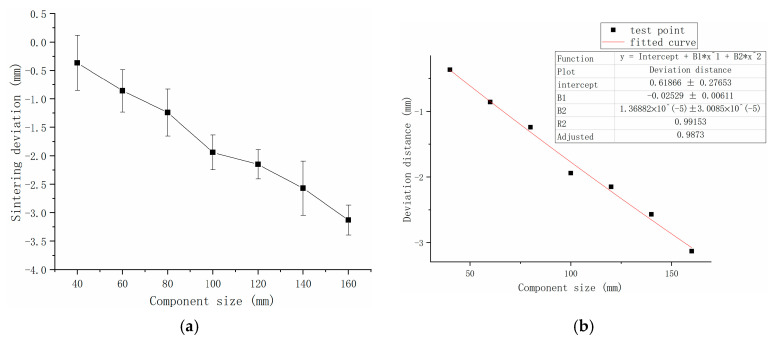
Dimensional deviation of molded parts: (**a**) deviation of base dimensions of formed parts; (**b**) formed part dimensional deviation fitting.

**Figure 16:**
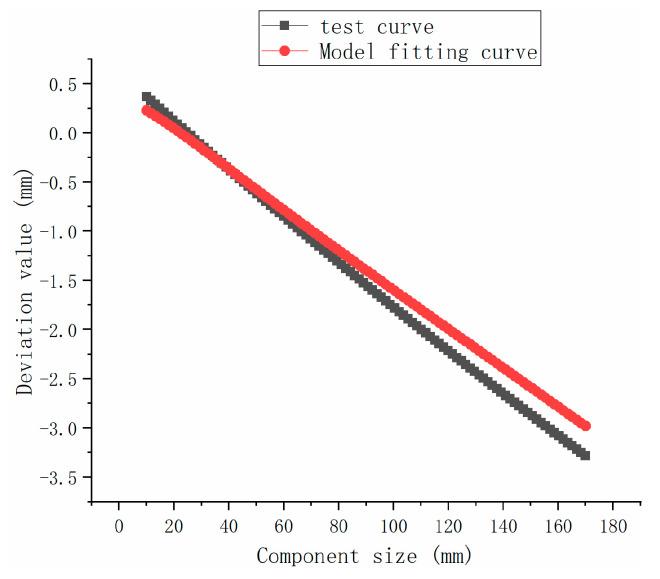
Comparison of the revised and experimental functions.

To make the proposed accuracy compensation model feasible, the relationship between the dimensional deviation term L_∆ and the input parameter L is curve fitted based on the printing results used to obtain Equation (13), and the fitting process is shown in Figure 15:

The aforementioned ${∆}_{Las}$, ${∆}_{T}$, ${∆}_{\rho }$, ${∆}_{SS}$, and four terms are substituted into Equation (1) to obtain Equation (14). The adjusted revision coefficients α = 1, β = 0.1235, γ = 0.555, and ω = 0.4 are input to Equation (15) to obtain the final compensation model. The effect of the relationship between the theoretical and the revised model dimensions is obtained from these coefficients (as shown in Figure 16):

(13) $$L_{∆}=0.61866-0.02529\cdot L+1.36882\times 10^{-5}\cdot L^{2}$$

(14) $$L_{∆}=-\left[{\omega \cdot ∆}_{Las}\right]-\left[{\alpha \cdot ∆}_{T}\right]-\left[{\beta \cdot ∆}_{\rho }\right]+\left[\gamma \cdot {∆}_{SS}\right]$$

(15) $$L_{∆}=-0.09381\omega -0.03797\omega L-0.001541\alpha L-\beta L\left(1-\sqrt[{3}]{\frac{C_{1}\times \left(0.62478+0.1614\times 0.95208^{L}\right)}{C_{0}\times 1.176}}\right)+0.723753\gamma$$

## 4. Verification of Proposed Accuracy Compensation Model

### 4.1. Non-Revised Base Size Forming Experiment

**Figure 17:**
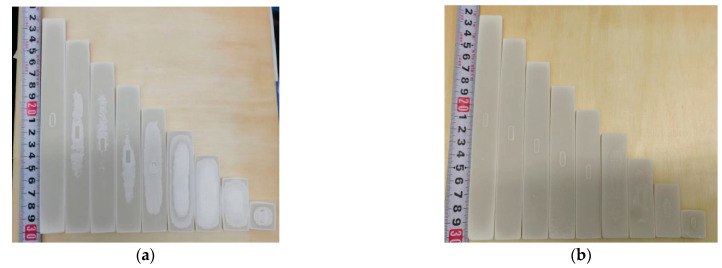
Selective laser sintering of molded parts: (**a**) before sandblasting; (**b**) after sandblasting.

The deformation of the laser-sintered fabricated parts on a planar scale can be characterized by non-revised base size sample forming experiments. The process parameters of the sintering experiments are shown in Table 5, and the dimensional parameters of the molded parts are shown in Table 6. Here, the sintered parts were scanned in 3D using a soft brush to remove the excess powder, and the sandblasted parts were scanned in 3D. The printing results are shown in Figure 17:

It is possible to determine the blasting process with a blowing pressure of 0.4 MPa for 100 mesh white corundum. Post-treatment of pure PES parts with 100 mesh white corundum 0.4 Mpa blowup pressure can be determined in order to effectively remove heat-affected areas without damaging the print. Therefore, for material of the same strength, the corresponding adjustment of the blowing pressure and the abrasive strength can effectively treat the surface, and it can simultaneously ensure a reasonable dimensional accuracy.

### 4.2. Experimental Verification of Compensation

**Figure 18:**
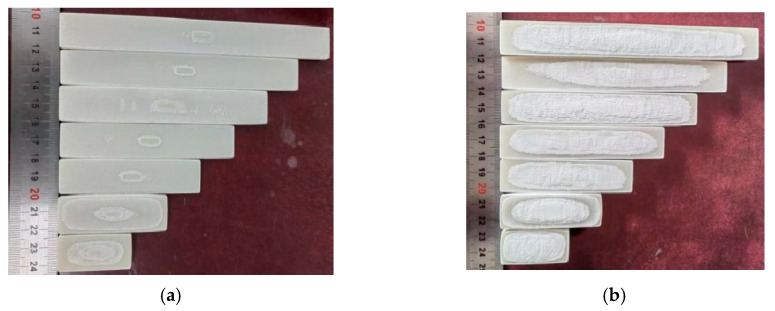
Selective laser sintered part after compensation: (**a**) before sandblasting compensating molding parts; (**b**) after sandblasting compensating molding parts.

**Figure 19:**
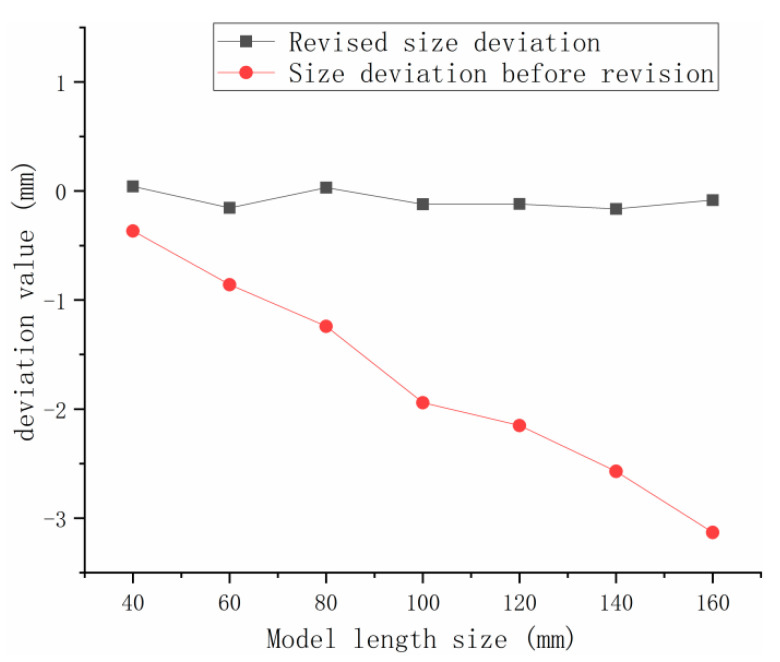
Dimensional deviation of the model before and after compensation.

**Figure 20:**
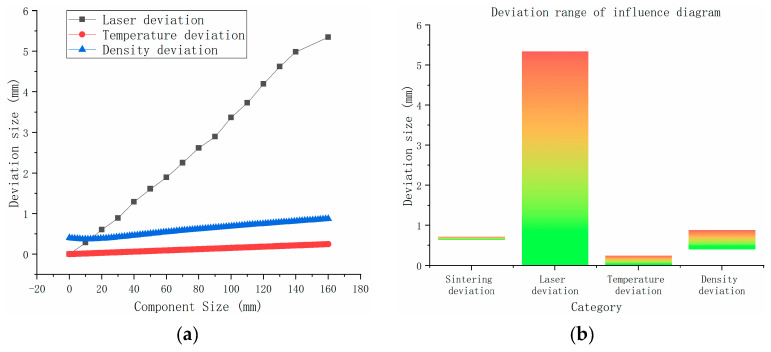
Comparison of deviation: (**a**) length comparison; (**b**) overall range comparison.

The print results after compensation using the proposed compensation model are shown in Figure 18. The overall mean value of the revised dimensional deviation of the molded parts is −0.08121 mm, the variance is 0.089121, the 0.95 confidence interval is [−0.30243, 0.14001], whilst the mean value of the deviation changed from −1.75117 mm before revision to −0.08121 mm and the deviation correction efficiency was approximately 95%. The dimensional deviations of the fabricated parts improved significantly (as shown in Figure 19). The dimensional accuracy grade of the model reached IT9–IT11 in the limit and fit standard (ISO286-1:1988) [32]. A comparison of the dimensional range affected by the combined laser reference deviation, the temperature deviation, the density deviation, and the secondary sintering deviation is shown in Figure 20. Under the same process parameters, the tensile strength of the mechanical sample can reach 6.7 Mpa, which accords with the basic mechanical properties of the material. 

## 5. Conclusions

(1) For the LS equipment, the influence of accuracy is ranked as follows: laser scanning deviation > sintering deviation ≥ density deviation > temperature deviation, among which the sintering deviation is relatively the most stable, and it can be considered as a constant. Laser deviation varies dramatically with the size of the component. Priority should be given to correcting laser scanning deviation when accuracy is considered. The influence of temperature deviation on the forming process of materials with a low preheating temperature is relatively small.
(2) The experimental method in this paper is aimed at the non-crystalline thermoplastic polymer material universal compensation method, which is the specific PES material as the prototype of the compensation model. Using the compensation model proposed in this paper, the effective rate of deviation correction is about 95%.
(3) The proposed accuracy compensation method can basically control the plane dimension of the laser-sintered solid parts within 0.165 mm to the accuracy level of IT9–IT11 of the limit and fit standard (ISO286-1:1988), which has a certain practical application value in the corresponding precision level application field.
(4) If crystalline polymer materials are considered, an additional term for the crystalline shrinkage function must be implemented in a revised model, which is equally valid after fitting the parameters using the experiments described in this paper.
(5) The proposed powder bed dimensional accuracy compensation model can be summarized into a process parameter library through a large number of experiments. This parameter library can be used to assist computer software in order to obtain an intelligent process system in order to improve the printing accuracy of the LS equipment.

## Figures and Tables

**Table 1:** Variables of the mathematical model.

Parameters	Description
L	Baseline dimensions (generated by a computerized digital model) are used to input the slicing software molding equipment for rapid prototype processing. Taking [L > 0], the theoretical value is specified to be greater than 0.
$L_{meas}$	The actual size of the part, which is generated by 3D scanning and mapping, or can be understood as the actual size obtained by printing. Taking [$L_{meas}$ > 0], the actual measured value of the part is specified to be greater than 0.
$L_{∆}$	Defined as the dimensional deviation term between the reference dimension L and the measured dimension L_meas.
${∆}_{Las}$	Laser reference deviation due to the optical path control accuracy, laser radiation accuracy, and other factors generated by the reference deviation. The value of ${∆}_{Las}$ is specified as the L–L grid, and the L grid is the measured value of the laser sintering calibration grid determined by the experiment in Section 2.3 below.
${∆}_{T}$	Temperature deviation. Due to the existence of large temperature variations in the molding process, a base temperature shrinkage deviation exists. Taking [${∆}_{T}$ > 0], which is estimated by the linear expansion coefficient, the actual process is a shrinkage term, which takes a positive value.
${∆}_{\rho }$	Density deviation. The presence of a large density change in the material from the powder stacked state to the solid state (which is also called sintering deviation). Taking [${∆}_{\rho }$ > 0], which is generated by the density change, the actual process is the shrinkage term, which takes a positive value in Equation (1).
${∆}_{SS}$	Take [${∆}_{SS}$ > 0], which is generated by temperature conduction and is an incremental term in the actual process, and takes a positive value in Equation (1). In the case of secondary sintering deviation, the material near the part is difficult to remove because it is denatured from the absorbed energy and thus adheres to the part.
α	Temperature deviation correction factor.
β	Density deviation correction factor.
γ	Revision factor for secondary sintering deviation.
ω	Revision factor for laser reference deviation.

**Table 2:** Functional parameters of LS equipment (CX_A200).

Projects	Parameters
Forming powder cylinder size	220 × 220 × 200 mm
Preheating temperature	≤100 °C
Layer thickness support	0.08–0.2 mm
Scanning speed	≤3000 mm/s
Spot size	0.4 mm
Laser power	40 W

**Table 3:** Functional parameters of Colin3D scanner device.

Project	Parameter
Working form	Fixed photo scanning measurement
Detection form	Blue light grating array
Recognition of depth of field	200 mm
Measurement accuracy	0.05 mm

**Table 4:** Powder supply powder stacking density $\rho_{0}$.

Item	1	2	3	4	5	6	7	Average
$\rho_{0}\;$ × 10^−3^ (g/mm^3^)	0.750	0.711	0.761	0.721	0.727	0.748	0.731	0.735

**Table 5:** Process parameters of sintering experiment.

Project	Parameters
Preheat time	30 min
Preheat temperature	89 °C
Scanning speed	2500 mm/s
Slicing thickness	0.1 mm
Powder supply multiplier	1.5
Laser power	15 W

**Table 6:** Dimension parameters of rectangular forming parts.

Project	Size (mm)
Length (X-direction)	20, 40, 60, 80, 100, 120, 140, 160, 180
Width (Y-direction)	20
Height (Z-direction)	5

## Data Availability

All relevant data are within the manuscript and its additional files.
